# The Role of Zn Ions in the Structural, Surface, and Gas-Sensing Properties of SnO_2_:Zn Nanocrystals Synthesized via a Microwave-Assisted Route

**DOI:** 10.3390/s24010140

**Published:** 2023-12-26

**Authors:** Luís F. da Silva, Mattia A. Lucchini, Ariadne C. Catto, Waldir Avansi Jr., Sandrine Bernardini, Khalifa Aguir, Markus Niederberger, Elson Longo

**Affiliations:** 1 Laboratory for Multifunctional Materials, Department of Materials, ETH Zürich, 8093 Zürich, Switzerland; mattia.a.lucchini@gmail.com (M.A.L.); markus.niederberger@mat.ethz.ch (M.N.); 2 Laboratory of Nanostructured Multifunctional Materials, Federal University of São Carlos, São Carlos 13565-905, Brazil; w_avansi@yahoo.com.br; 3 Center for the Development of Functional Materials, Federal University of São Carlos, São Carlos 13565-905, Brazil; ade.catto@gmail.com (A.C.C.); elson.liec@gmail.com (E.L.); 4 Aix Marseille Univ, CNRS, IM2NP, 13397 Marseille, France; sandrine.bernardini@im2np.fr (S.B.); khalifa.aguir@im2np.fr (K.A.)

**Keywords:** SnO_2_, zinc, non-aqueous synthesis, microwave route, local structure, surface properties, chemoresistors

## Abstract

Although semiconducting metal oxide (SMOx) nanoparticles (NPs) have attracted attention as sensing materials, the methodologies available to synthesize them with desirable properties are quite limited and/or often require relatively high energy consumption. Thus, we report herein the processing of Zn-doped SnO_2_ NPs via a microwave-assisted nonaqueous route at a relatively low temperature (160 °C) and with a short treatment time (20 min). In addition, the effects of adding Zn in the structural, electronic, and gas-sensing properties of SnO_2_ NPs were investigated. X-ray diffraction and high-resolution transmission electron microscopy analyses revealed the single-phase of rutile SnO_2_, with an average crystal size of 7 nm. X-ray absorption near edge spectroscopy measurements revealed the homogenous incorporation of Zn ions into the SnO_2_ network. Gas sensing tests showed that Zn-doped SnO_2_ NPs were highly sensitive to sub-ppm levels of NO_2_ gas at 150 °C, with good recovery and stability even under ambient moisture. We observed an increase in the response of the Zn-doped sample of up to 100 times compared to the pristine one. This enhancement in the gas-sensing performance was linked to the Zn ions that provided more surface oxygen defects acting as active sites for the NO_2_ adsorption on the sensing material.

## 1. Introduction

Climate change has been the main topic covered in scientific forums to find ground-breaking strategies to minimize the influence of human activities on global environmental problems. Industrial activities and the burning of fossil fuels have been primarily responsible for the release of various gases into the atmosphere [1,2,3,4]. Some of these gaseous species, in addition to contributing to the greenhouse effect, are also harmful to human health, such as NO_2_, O_3_, and CO [2,5,6]. Unfortunately, people who live and/or work near these pollutants are more likely to contract certain diseases, such as respiratory and cardiovascular ones, and even cancer. Recently, the World Health Organization (WHO) reported that atmospheric pollution has been responsible for the death of approximately 7 million people worldwide per year [2,4,7].

Nitrogen dioxide (NO_2_) is a toxic gas, and exposure to it can cause inflammation of the airways, asthma, and other respiratory sicknesses (e.g., coughing and difficulty in breathing) [6,8,9]. The largest sources of NO_2_ gas emissions are mainly linked to the burning of fossil fuels [1,10,11]. The United States Environmental Protection Agency (US EPA) recommends a NO_2_ level of 0.1 parts per million (ppm) for a maximum of 1 h of exposure [8]. For instance, the air pollution levels in Europe have been reduced over the last few decades; even so, NO_2_ gas has been responsible for thousands premature deaths every year [12].

Gas sensors, particularly chemoresistors based on SMOx NPs (semiconductor metal oxide nanoparticles) detect NO_2_ through a change in electrical resistance when exposed to the gas. When NO_2_ interacts with the surface of the sensor, it causes a change in the conductance of the semiconductor metal oxide, leading to a measurable change in resistance. This change is then converted into an electrical signal, allowing for the detection and quantification of NO_2_. The sensors are designed to be sensitive, selective, and operational at room temperature, making them suitable for various applications such as environmental monitoring and industrial safety [13,14]. They are designed to provide accurate and reliable monitoring of NO_2_ levels, with a wide range of detection capabilities, from parts per billion (ppb) to parts per million (ppm).

These data reveal the need for the development of efficient sensing devices that could make the monitoring of air pollution levels more accessible and reliable, especially for detecting sub-ppm levels. Among the devices applied for the detection of hazardous gas, resistive “chemoresistors” are gas sensors which have been used to detect a variety of analytes, such as NO_2_ gas. Gas sensors based on SMOx NPs have been outstanding due to their high sensing activity, linked to their high-surface area that favors the catalytical processes between the SMOx surface and analyte [1,15,16,17,18,19].

Among the traditional SMOx, tin dioxide (SnO_2_) is one of the most studied sensing materials due to its sensitivity to a variety of analytes [20,21,22,23]. It is an n-type SMOx with an energy gap of ca. 3.6 eV (at 300 K) which makes it also promising for photocatalysis and energy generation [24,25,26,27,28,29].

One well-known way to improve the sensing properties of SnO_2_ is to add certain dopants, such as transition metals and noble metals [16,30,31,32,33,34,35]. Previous studies have demonstrated that an extrinsic doping/co-doping strategy has a significant influence on the sensing performance (e.g., sensitivity, selectivity, and working temperature) [16,17,30,36,37]. Vallejos and co-workers prepared Au-decorated SnO_2_ nanorods via aerosol-assisted chemical vapor deposition to be applied as gas sensors. The authors found an enhancement in the sensitivity and selectivity toward H_2_ compared to the bare sample [30]. In a previous study, we observed an increase in ozone sensor response of ZnO thin films with Co content. This enhancement in the gas-sensing properties was attributed to the presence of oxygen vacancies, which facilitated ozone adsorption [17].

Concerning the effect of Zn addition on the gas-sensing performance of SnO_2_, some investigations have reported promising data, as illustrated in Table 1. These studies have reported that the addition of Zn ions to SnO_2_ may modify its electronic structure, increasing the surface oxygen defects due to charge compensation, which may affect the sensing performance [25,37,38].

Regarding the synthesis method, nonaqueous routes have attracted attention in terms of obtaining materials with diverse functional properties in a controlled and reproductive way [44,45]. The microwave-assisted nonaqueous synthesis of organic solvents under the exclusion of water is able to overcome some of the major limitations of aqueous systems. The main advantages of this route are the simplicity, short synthesis times (few minutes), and relatively low temperatures (<200 °C) [45,46], mainly when compared to the data in Table 1. In the gas-sensing area, we have previously reported the potential of the microwave-assisted route to obtain ZnO/SnO_2_ nanoheterostructures applied as ozone gas sensors. The experiments demonstrated that heterostructures were promising for detecting ozone gas under UV-light stimulation [47].

Motivated by these considerations, we conducted a detailed investigation of the effects of Zn ions on the structural, surface, and NO_2_ gas-sensing properties of SnO_2_ NPs synthesized via the microwave-assisted nonaqueous method. The properties of Zn-doped SnO_2_ NPs were studied by X-ray diffraction (XRD), X-ray absorption near-edge structure spectroscopy (XANES), high-resolution transmission electron microscopy (HRTEM), and X-ray photoelectron spectroscopy (XPS). The gas-sensing experiments demonstrated good sensing activity at a mild temperature (150 °C), which could further be improved by increasing the Zn content. In addition, the Zn-doped nanocrystals revealed high stability of their NO_2_ sensing performance under different humidity levels.

## 2. Experimental

### 2.1. Reagents

Zn (II) acetylacetonate hydrate (99.995%), Sn (IV) chloride (99.99%), toluene (99%) benzyl alcohol (99.8%), acetylacetonate (99.5+%), hexyl alcohol (98%), and 1,3-propanediyl (99.5+%) were all purchased from Aldrich and used as-received without further purification.

### 2.2. Synthesis Procedure

Pristine SnO_2_ and Zn-doped SnO_2_ NPs (Zn/(Zn + Sn) = 5 and 30 mol %) were synthesized using a microwave-assisted nonaqueous method in a CEM Discover reactor operating at a frequency of 2.45 GHz. The synthesis of the Zn-doped SnO_2_ samples was performed by adding 12.3 mmol of tin tetrachloride and zinc acetylacetonate to 10 mL of toluene and then 30 mL of benzyl alcohol was added to the solution. Concerning the undoped SnO_2_ sample, the procedure was similar except for the addition of a zinc precursor. Thereafter, the reaction mixture was transferred into a 35 mL glass tube and sealed with a Teflon cap. The microwave-assisted treatment was performed for 20 min at 160 °C, maintaining the reaction mixture in continuous stirring. The precipitate was separated from the liquid phase by centrifugation, washed three times with ethanol, and then dried overnight at 60 °C. The Zn-doped samples were labeled as SnZn1 (5 mol % Zn) and SnZn2 (30 mol % Zn).

### 2.3. Characterization Techniques

X-ray diffraction (XRD) patterns were measured using a Shimadzu XRD 6100 (Shimadzu Corporation, Kyoto, Japan) diffractometer with a monochromatic Cu Kα source, collected at room temperature, in a continuous scan mode with a speed of 2° min^−1^ and a step of 0.02°. The morphological properties were analyzed using a field emission scanning electron microscope (FE-SEM, Supra35, ZEISS Company, Jena, Germany) and a high-resolution transmission electron microscope (HRTEM, TECNAI G2 F30, FEI Company, Hillsboro, OR, USA) operating at 200 kV. The mean crystal size was estimated from the analysis of the TEM images through the measurement of approximately 100 NPs. X-ray absorption near-edge spectroscopy (XANES) experiments were carried out at the Brazilian Synchrotron Light Laboratory. The experiments were carried out at the Zn K- and Sn-L3 edges at room temperature using a flat Si(111) double crystal monochromator. The processing and analysis of XANES spectra were performed using the MAX software package [48]. XPS analysis was performed on a Thermo Scientific K-Alpha spectrometer using a monochromatic Al Kα source. The as-obtained data were analyzed using Casa XPS software 2.3.25 (Casa Software Ltd., Teignmouth, Devon, UK), and the spectra were calibrated using the C 1s line (284.8 eV) of the adsorbed carbon on the sample surface.

### 2.4. Gas-Sensing Experiments

The detailed procedure of the preparation of sensing devices is described in the Supporting Information. The gas-sensing experiments were carried out in a dynamic chamber that allowed us to control the operating temperature and the gas concentrations by using a set of mass flow controllers. Our workbench allowed exposure of the samples to distinct NO_2_ levels, ranging from 0.1 to 2.0 ppm. The total gas flow was kept equal to 500 SCCM. The applied DC voltage was kept constant (1 V), while the electrical resistance was monitored using a sourcemeter (model 2540, Keithley Instruments, Cleveland, OH, USA). Further information can be found in our previous papers [17,49,50]. The sensor response (S) was estimated following the procedure reported elsewhere [51].

The sensing performance of the Zn-doped nanocrystals in ambient moisture and with a reducing gas (here, CO gas) was investigated in another chamber (piezo-driven probe model, NEXTRON company; Busan, Republic of Korea). This chamber was connected to a system that allowed the control of temperature, relative humidity (% RH; from 0 to 40%), and target gas concentration. For the sake of comparison, the performance of the sample with NO_2_ and CO gases was evaluated under the same conditions (e.g., sensing chamber, %RH, operating temperature, and gas flow).

## 3. Results and Discussion

### 3.1. Characterization of Zn-Doped SnO_2_ Nanocrystals

**Figure 1:**
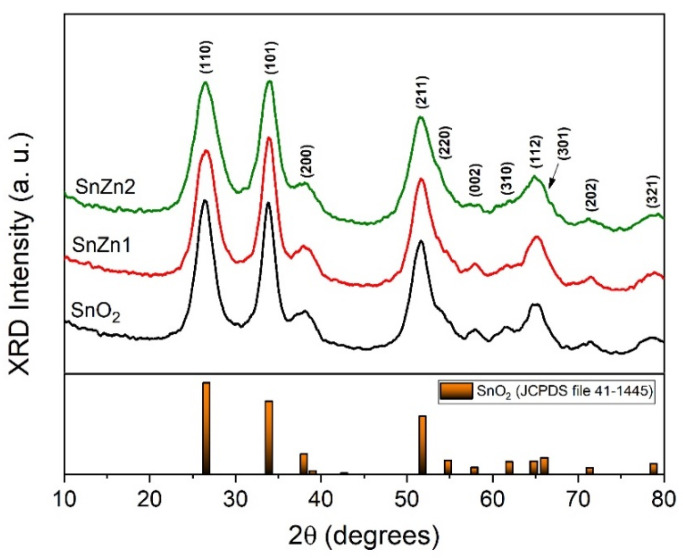
XRD patterns of the undoped and Zn-doped SnO_2_ NPs. At the bottom, the vertical bars correspond to the data obtained from the JCPDS file #41-1445.

XRD patterns of the doped and undoped SnO_2_ samples are presented in Figure 1. All reflections were indexed to a tetragonal SnO_2_ phase, in accordance with JCDPS file #41-1445. It should be mentioned that no peaks related to Zn-rich phases were identified, indicating that Zn ions were homogenously incorporated into the SnO_2_ lattice. Furthermore, the slight broadening of the XRD peak with increasing Zn content can be linked to the reduction in crystallographic domains. The average crystallite size was estimated from the FWHM of the (110) XRD peak using Scherrer’s equation, and the obtained values were 3.2 nm (SnO_2_), 3.0 nm (SnZn1), and 2.6 nm (SnZn2). Wang and co-workers observed a similar tendency for Zn-doped SnO_2_ nanostructures obtained via a hydrothermal treatment, attributing this peak enlargement to the reduction in crystallinity and crystal size [37].

**Figure 2:**
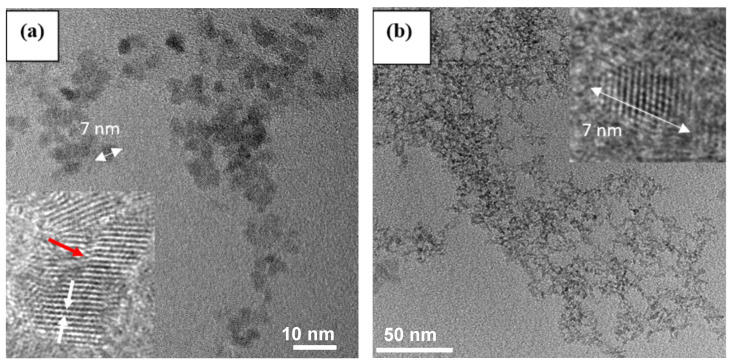
TEM images of selected samples: (**a**) pristine SnO_2_ and (**b**) SnZn2 NPs. Inset shows the HRTEM images of their respective samples. (Left) White arrows indicate the atomic distance, and red arrow the coalescence between particles. (Right) White arrows indicate the nanocrystal size.

Figure 2 presents the TEM analysis of pristine SnO_2_ and SnZn2, where it can be observed that the spherical morphology of both samples was quite similar, even for the Zn-doped sample, with an average crystal size of 7 nm. HRTEM images, the inset of the respective TEM images, show that the distance between the planes was about 0.34 nm, corresponding to the (110) crystallographic plane of the tetragonal SnO_2_, in accordance with the XRD data. The HRTEM images present agglomerated NPs, indicating a coalescence between particles (illustrated by a red arrow) which can be related to the crystal growth mechanism [52,53,54]. This is expected as previous studies have reported that the oriented attachment mechanism plays an important role in SnO_2_ synthesis in hydrothermal conditions [52,55].

**Figure 3:**
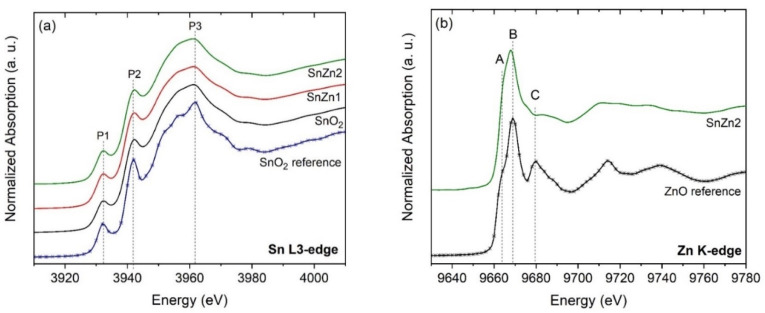
XANES spectra of undoped and Zn-doped SnO_2_ NPs. (**a**) Sn L3- and (**b**) Zn-K edge. For the sake of comparison, the spectra of commercial samples are inserted in panels (**a**,**b**).

To investigate the electronic structure of the short-range order of Zn-doped SnO_2_ NPs, XANES spectra were collected at the Sn-L3 and Zn-K edges, as displayed in Figure 3. For the sake of comparison, commercial micrometric powders (ZnO and SnO_2_) were used as references. XANES spectra at the Sn-L3 edge present three electronic transitions (labeled as P1, P2, and P3), as shown in Figure 3a. The physical origin of these peaks corresponds to electronic transitions from 2p_3/2_ to 5s_1/2_ and *n*d_5/2_ levels [28,56]. It can be seen that the pristine SnO_2_, SnZn1, and SnZn2 spectra are quite similar to each other, presenting less significant oscillations compared to the reference sample. This feature reveals that these samples have a low short-range order around the Sn atoms that can be attributed to the rapid crystallization provided by the microwave-assisted route. Regarding the Zn addition, no significant influence on the short-range structure of the SnO_2_ samples was observed within the zinc content evaluated here.

Figure 3b illustrates the Zn K-edge spectra of SnZn2 and the reference ZnO. Three electronic transitions are visible in the spectra, labeled as A, B, and C. Regarding the electronic transitions A and B, they are attributed to the 1s to 4p transition, and the C transition is due to multiple scattering contributions [17,57]. The analysis of both spectra showed that they differ from each other, especially in the post-edge region between 9670 and 9780 eV. This means that the chemical environment of Zn atoms in SnO_2_ samples is not the same as in ZnO, suggesting their homogeneous incorporation in the SnO_2_ network. Thus, XANES and XRD analyses allow us to affirm that the addition of Zn ions did not favor the clustering of ZnO (at both long-and short-range) in the Zn-doped SnO_2_ nanocrystals.

XPS analysis was carried out to further characterize the pristine SnO_2_ and Zn-doped SnO_2_ samples and illustrate their surface compositions and electronic states. In the XPS survey spectra of these samples, Figure S2a, the Sn and O were found to be the predominant elements, whilst the Zn was present only in the doped samples, as expected. The quantification of peaks from survey spectra revealed that the Zn atom percentages (at%) were 0.6% and 2.0% for the SnZn1 and SnZn2 samples.

Figure S2b shows the Sn 3d high-resolution XPS spectra, in which the Sn 3d_5/2_ and Sn 3d_3/2_ peaks were located at 486.6 and 495.0 eV, confirming the presence of Sn^4+^ in all samples. No significant shift in the Sn 3d_5/2_ and Sn 3d_3/2_ peaks positions with Zn addition was observed, indicating that the zinc addition did not affect the local electronic structure of Sn. From the high-resolution Zn 2p XPS spectra of the Zn-doped SnO_2_ samples, Figure S2c, two symmetrical peaks were identified at binding energies of 1022.7 and 1045.8 eV, with a spin-orbit splitting of 23.1 eV, corresponding to Zn 2p_3/2_ and Zn2p_1/2_ [58].

**Figure 4:**
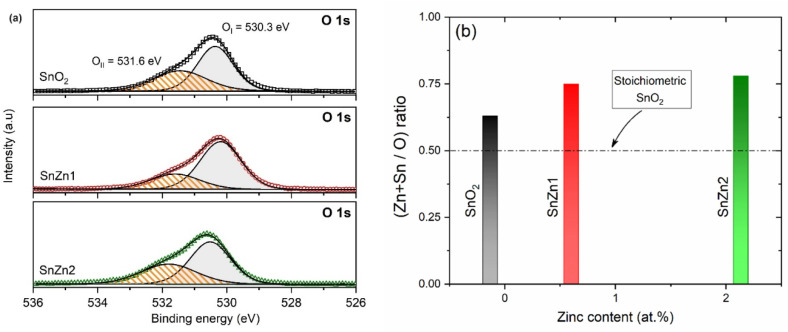
O1 s XPS spectra of the pristine SnO_2_, SnZn1, and SnZn2 NPs. (**a**) High-resolution scan and (**b**) variation in (metal/oxygen) ratio as a function of zinc content.

Regarding the high-resolution O 1s spectra, Figure 4a, a very asymmetric peak was found that indicates the presence of different oxygen species. These spectra were deconvoluted into two Gaussian–Lorentzian components, labeled as O_I_ and O_II_. The main component at 530.2 eV (O_I_) was typical of lattice oxygen anions bound to the metal cations, whilst the second component (O_II_) was linked to hydroxyls and adsorbates [23,37,59,60]. From the O 1s high-resolution spectra, the relative percentage area of the O_II_ component was approximately 38.7% (SnO_2_), 39.5% (SnZn1), and 49.3% (SnZn2), indicating that Zn addition increased slightly the concentration of hydroxyls on the surface of the samples. In addition, the metal-to-oxygen ratios (metal/oxygen) of the pristine and Zn-doped samples were also estimated from survey spectra only considering the metal–oxygen bond in O 1s spectra (530.2 eV), and the obtained data are displayed in Figure 4b. The analysis of this figure reveals a tendency to increase the metal/oxygen ratio with zinc content. Note that a stoichiometric sample (SnO_2_) must present a metal/O ratio of 0.5, respectively. Thus, the behavior observed in Figure 4b reveals that samples prepared via the microwave-assisted nonaqueous route are oxygen deficient, and the zinc addition into the SnO_2_ network favors the enhancement of surface oxygen defects, here induced by the partial substitution of Zn^2+^ by Sn^4+^ ions. Wang and co-workers using experimental and theoretical approaches also demonstrated that the increase in Zn content favored the formation of oxygen vacancies in SnO_2_ nanostructures [37].

### 3.2. Gas-Sensing Measurements

**Figure 5:**
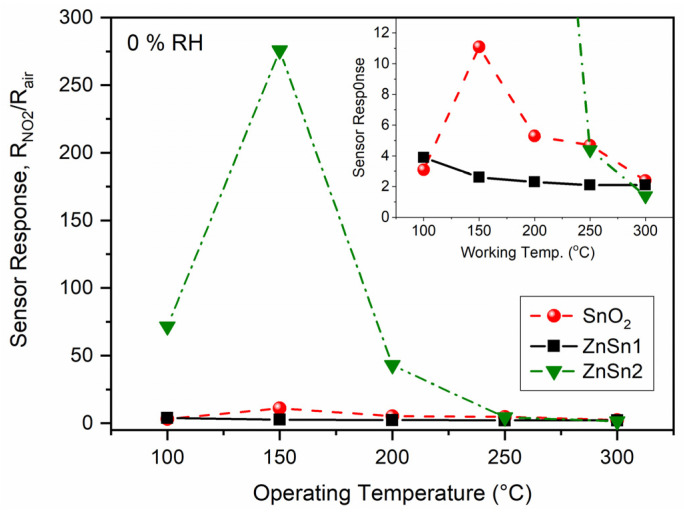
Sensor response of pristine and Zn-doped SnO_2_ NPs exposed to 1 ppm NO_2_ at different working temperatures. The inset shows in detail the response of SnO_2_ and SnZn1 samples. All measurements were performed under dry air (0% RH).

The gas-sensing performances of the pristine SnO_2_, SnZn1, and SnZn2 samples were evaluated for NO_2_ gas. To find the best working temperature of the samples, they were exposed to 1 ppm of NO_2_ with a heating temperature under the sensors varying from 100 to 300 °C, Figure 5. From the analysis of the curves, the highest responses were achieved at around 150 °C for the Zn-doped samples. At this temperature, the response of the SnZn2 sample was around 25 times higher than that of SnZn1 and 100 times higher than the pristine sample. The observed order of sensing performance was SnZn2 > SnZn1 > SnO_2_, demonstrating that the Zn addition improved the sensitivity to NO_2_ gas. This enhancement can be linked to the addition of Zn ions into the SnO_2_ nanocrystals which favored the formation of surface oxygen defects, as above-mentioned. Many studies have highlighted the importance of oxygen defects for improving the sensitivity of SMOx [25,61,62,63]. Thus, both Zn ions and surface oxygen defects exert a positive influence on the sensing activity of SnO_2_ NPs, acting as active sites for NO_2_ sensing reactions. Some researchers have reported that nanosized features and the presence of unsaturated cations and oxygen defects may facilitate the interaction of the analyte at the MOX surface, thus increasing its sensitivity [16,64,65].

**Figure 6:**
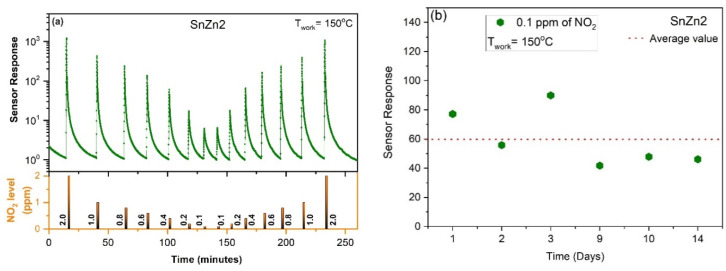
(**a**) Gas-sensing performance of SnZn2 at 150 °C exposed to 0.1 to 2.0 ppm of NO_2_ gas. (**b**) Long-term stability of this sample upon exposure to 0.1 ppm of NO_2_ gas for a period of approximately 2 weeks. All measurements were performed under dry air (0% RH).

Considering the superior performance of SnZn2 to detect NO_2_ gas, it was further investigated at different NO_2_ levels ranging from 0.1 to 2 ppm at a working temperature of 150 °C. Figure 6a reveals that this sample was sensitive to all of these levels, with no evidence of saturation. It should be mentioned that these results demonstrate the practical feasibility of Zn-doped SnO_2_ NPs as a sensing material since NO_2_ gas is harmful to human health in concentrations higher than 0.1 ppm [8,66]. This figure also depicts the good repeatability of the response and recovery of the SnZn2 sample which was able to detect low concentrations even after consecutive exposure cycles.

The long-term stability of SnZn2 was also evaluated, exposing it repeatedly to 0.1 ppm of NO_2_ gases over 14 days at an operating temperature of 150 °C, as seen in Figure 6b. It can be noted that the sample was able to detect NO_2_ gas over the whole period, revealing that its surface remains active after several exposure cycles.

**Figure 7:**
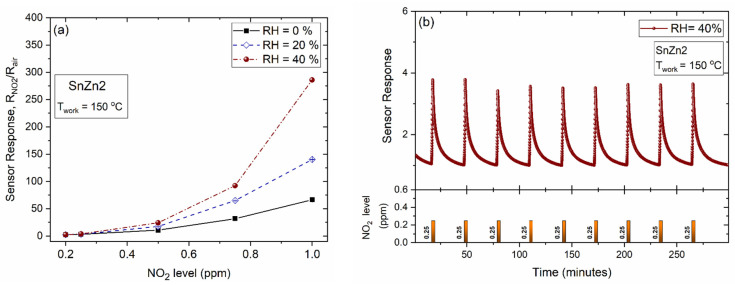
Sensor response of SnZn2 at 150 °C (**a**) exposed to different NO_2_ levels under different relative humidity and (**b**) exposed to a sequence of cycles of 0.25 ppm of NO_2_ under 40% RH.

The influence of relative humidity on the NO_2_ sensing performance was also investigated. To this end, the SnZn2 NPs were kept at 150 °C and then exposed to 0.2, 0.25, 0.5, 0.7, and 1 ppm of NO_2_ gas under different relative humidity values (0, 20, and 40% RH). It can be seen that the NO_2_ sensor response was enhanced with increasing relative humidity, as shown in Figure 7a. Note that the sample exhibited high repeatability of its response, confirming its reliability even under ambient moisture, as demonstrated by the nine exposure cycles displayed in Figure 7b.

According to the findings, the sensor response of SnZn2 to 1 ppm NO_2_ increased from S = 66.4 ± 0.4 (RH = 0%) to S = 285.9 ± 0.4 (RH = 40%). A low interference of humidity on the sensing performance is a desirable characteristic in the development of sensing materials [16,66,67,68,69,70]. Several investigations demonstrate that the presence of moisture in the ambient may impair the sensing activity [51,68,71,72]. In 2021, we reported that the sensing response of α-Fe_2_O_3_ to BTEX gases (Benzene, Toluene, Ethylbenzene, and Xylenes) was reduced as a function of humidity level [51]. This negative effect was linked to the competition of water and BTEX molecules for the same surface-active sites [51]. In contrast, Yan and co-workers found that humidity can improve the NO_2_ sensing performance of WS_2_/graphene composites working at room temperature [66]. Shooshtari and co-workers observed an increase in the ethanol sensing response of TiO_2_ nanowires for RH of up to 50% [73]. They explained that this behavior was a result of the presence of hydroxyl groups and oxygen adsorbates. For higher humidity levels (>50% RH), the hydroxyl groups cover almost all of the TiO_2_ surface, thus limiting the oxygen adsorption and consequently reducing the sensitivity toward ethanol [73].

**Figure 8:**
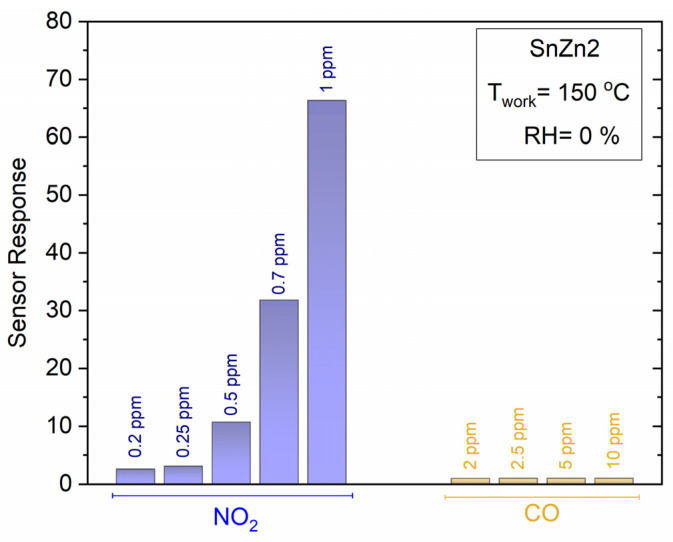
Comparison of the sensor response of SnZn2 exposed to NO_2_ (0.2 to 1 ppm) and CO (2 to 10 ppm). These measurements were performed at 150 °C in a dry air atmosphere (0% RH).

The SnZn2 sample was also evaluated to detect a reducing analyte, specifically CO gas, using the optimal temperature of 150 °C. Figure 8 shows that the same sample exhibited a very low sensitivity to CO gas. Despite detecting the CO gas, the sensing response of SnZn2 NPs toward NO_2_ was superior compared to CO gas, as seen in Figure 8.

It is clear that the results here obtained revealed that the addition of Zn ions into the SnO_2_ nanocrystals significantly enhanced the sensing performance toward sub-ppm NO_2_ levels. Nevertheless, it would be interesting in future works to find rational strategies to reduce the influence of moisture on gas sensitivity and to obtain a fair sensing performance (e.g., complete recovery, stability, and reproducibility) at working temperatures closer to room temperature.

## 4. Conclusions

We report here a versatile approach for preparing Zn-doped SnO_2_ NPs via a microwave-assisted nonaqueous route for use as promising chemoresistive NO_2_ sensors. XRD, XANES, and HRTEM analyses confirmed the presence of nanocrystalline SnO_2_ and the absence of spurious phases. Sn-L edge XANES spectra revealed a local disorder around Sn atoms caused by the rapid crystallization provided by the microwave-assisted route. Zn-K edge XANES indicated that the Zn ions were incorporated into the SnO_2_ network. XPS analyses revealed an increase in the oxygen defects at the SnO_2_ surface, probably due to the partial replacement of Sn^4+^ by Zn^2+^ ions. The presence of these defects was linked to the high sensor response of SnZn2 (2 at% of Zn) toward NO_2_ gas which was able to detect sub-ppm levels, i.e., from 0.1 ppm. Furthermore, the addition of Zn ions increased the moisture tolerance of SnO_2_ NPs, presenting a higher sensing response in a more humid environment (40% RH) compared to a dry one. The enhanced sensor properties were linked to the presence of Zn ions that favored the formation of surface oxygen vacancies, thus contributing to the adsorption of NO_2_ molecules. These findings highlight the promising properties of the microwave-assisted nonaqueous route for obtaining Zn-doped SnO_2_ NPs for chemoresistive NO_2_ sensors in practical applications.

## Figures and Tables

**Table 1:** Gas-sensing performance of Zn-doped SnO_2_ synthesized by different methodologies.

Synthesis Method	Temperature/Time of Synthesis	Target Gas	Gas Level Detected * (ppm)	Working Temperature (°C)	Reference
Hydrothermal	180 °C/16 h	Glycol	5.0	240	[38]
Spray pyrolysis	350 °C/**	Formic Acid	50.0	350	[39]
Hydrothermal	200 °C/12 h	Ethanol	40.0	250	[40]
Precipitation	550 °C/2 h	Ethanol	2.0	240	[41]
Hydrothermal	180 °C/24 h	Triethylamine	100.0	70	[37]
Hydrothermal	190 °C/ 12 h	NO_2_	1.0	350	[42]
Hydrothermal	140 °C/24 h	NO_2_	0.35	200	[43]
Microwave-assisted	160 °C/ 20 min	NO_2_	0.10	150	Present study

* Minimum gas level, experimentally detected/** deposition time not provided.

## Data Availability

Data are contained within the article and supplementary materials.

## Supplementary Materials

The following supporting information can be downloaded at: https://www.mdpi.com/article/10.3390/s24010140/s1, Figure S1: (a) Photograph of sensing platforms based on SnO_2_ and SnZn2 samples compared to a U.S one tenth-dollar coin. (b) FESEM image of the SnZn2 sample onto sensing platform.; Figure S2: XPS spectra of the Zn-doped SnO_2_ NPs synthesized via microwave-assisted route. (a) Survey scan, (b) Sn 3d and, (c) Zn 2p; Figure S3: NO_2_ gas-sensing performance of SnZn2 nanocrystals kept at 150 °C and exposed to different relative humidity levels. (a) 0 %RH, (b) 20 %RH, and (c) 40 %RH. Ref. [74] is cited in S1. Preparation of sensing devices.
