# Supplementary material for: The Role of Zn Ions in the Structural, Surface, and Gas-Sensing Properties of SnO2:Zn Nanocrystals Synthesized via a Microwave-Assisted Route
Source: Sensors (Basel). 2023 Dec 26;24(1):140. doi: 10.3390/s24010140 (PMC10781226; doi:10.3390/s24010140)
Supplement: Supplementary file 1 [file sensors-24-00140-s001.zip › sensors-2744255-supplementary.pdf]

Supplementary Material

The Role of Zn Ions in the Structural, Surface, and Gas-Sensing Properties of SnO_2:Zn Nanocrystals Synthesized via a Microwave-Assisted Route

Luís F. da Silva [1,2,*], Mattia A. Lucchini [1], Ariadne C. Catto [3], Waldir Avansi, Jr. [2], Sandrine Bernardini [4], Khalifa Aguir [4], Markus Niederberger [1] and Elson Longo [3]

[1] Laboratory for Multifunctional Materials, Department of Materials, ETH Zürich, 8093 Zürich, Switzerland; mattia.a.lucchini@gmail.com (M.L.); markus.niederberger@mat.ethz.ch (M.N.)
[2] Laboratory of Nanostructured Multifunctional Materials, Federal University of São Carlos, São Carlos 13565-905, Brazil; w_avansi@yahoo.com.br
[3] Center for the Development of Functional Materials, Federal University of São Carlos, São Carlos 13565-905, Brazil; ade.catto@gmail.com (A.C.C.); elson.liec@gmail.com (E.L.)
[4] Aix Marseille University, CNRS, IM2NP, Marseille, France; sandrine.bernardini@im2np.fr (S.B.); khalifa.aguir@im2np.fr (K.A.)
* Correspondence: lfsilva83@gmail.com

S1. Preparation of sensing devices

The sensing devices were fabricated via doctor blading of the NPs onto a Si/SiO_2 substrate containing 100 nm thick Pt electrodes separated by a distance of 50 μm. The procedure for the slurry preparation was adapted from the literature.[1] First, 100 mg of the as-obtained SnO_2:Zn powders were mixed in a solution with 100 μL of propanedyl, 900 μL of hexyl alcohol, and 90 μL of acetylacetone. The mixture was sonicated and 500 mg of Pluronic was added, and then it was stirred during 12 hours. The Zn-doped SnO_2 thick films were prepared by the doctor blade method by depositing 1 layer of the obtained slurry onto Si/SiO_2 substrates containing Pt electrodes. The speed of the blade was set to 15 mm s^{-1} and its height was set to +75 μm towards the sensing platform. The as-prepared films were annealed in an electric oven under an air atmosphere to eliminate the residual organics coming from the slurry. Fig. S1 shows photograph of two representative samples (SnO_2 and SnZn2) deposited onto the sensing platforms.

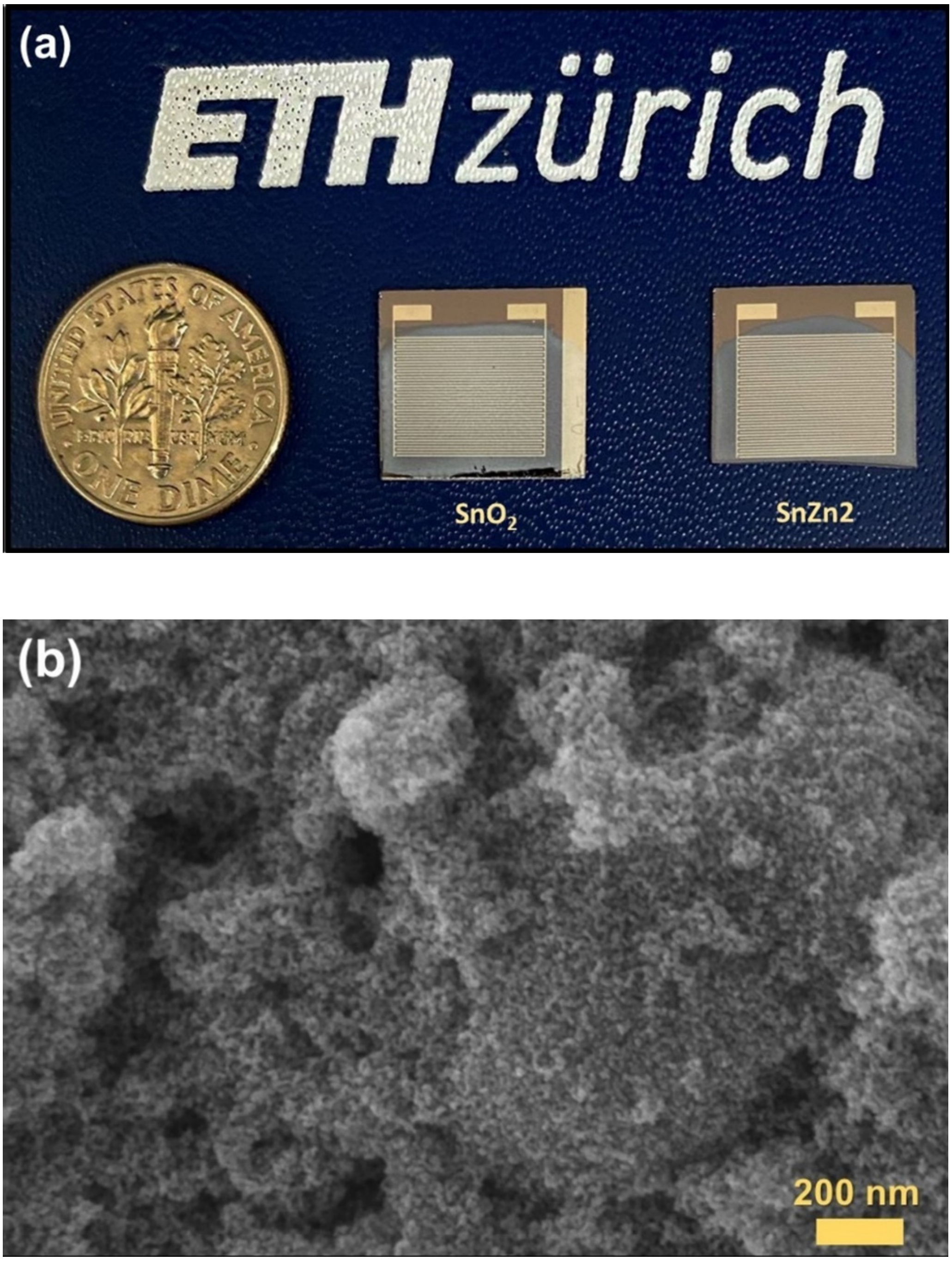

Figure S1. (a) Photograph of sensing platforms based on SnO_2 and SnZn2 samples compared to a U.S one tenth-dollar coin. (b) FESEM image of the SnZn2 sample onto sensing platform.

S2. XPS analysis

Figure S2 shows the XPS survey spectra of the pristine SnO_2, SnZn1, and SnZn2 samples synthesized via microwave-assisted method.

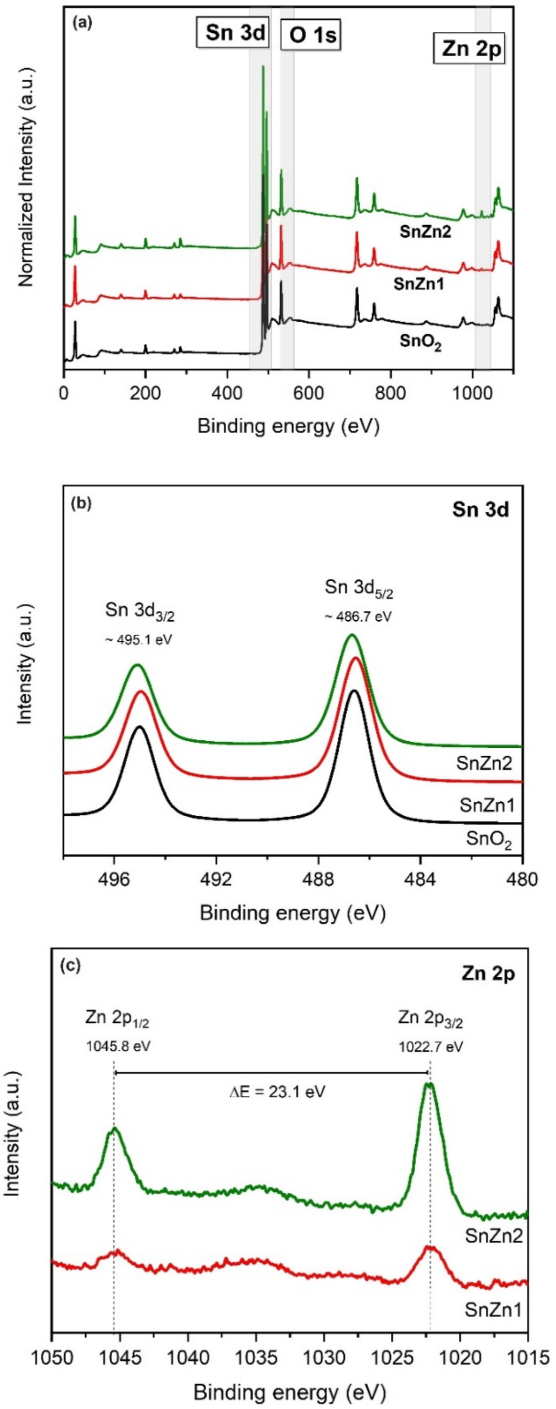

Figure S2. XPS spectra of the Zn-doped SnO_2 NPs synthesized via microwave-assisted route. (a) Survey scan, (b) Sn 3d and, (c) Zn 2p.

S3. Gas-sensing tests

Figure S3 shows the sensing performance of SnZn2 nanocrystals exposed to NO_2 levels (0.2 to 1.0 ppm) at an operating temperature of 150°C and under different humidity levels (0 %RH, 20 %RH, and 40 %RH). The y-axis (sensor response) range was the same to provide a good visualization of the sensing performance of the samples.

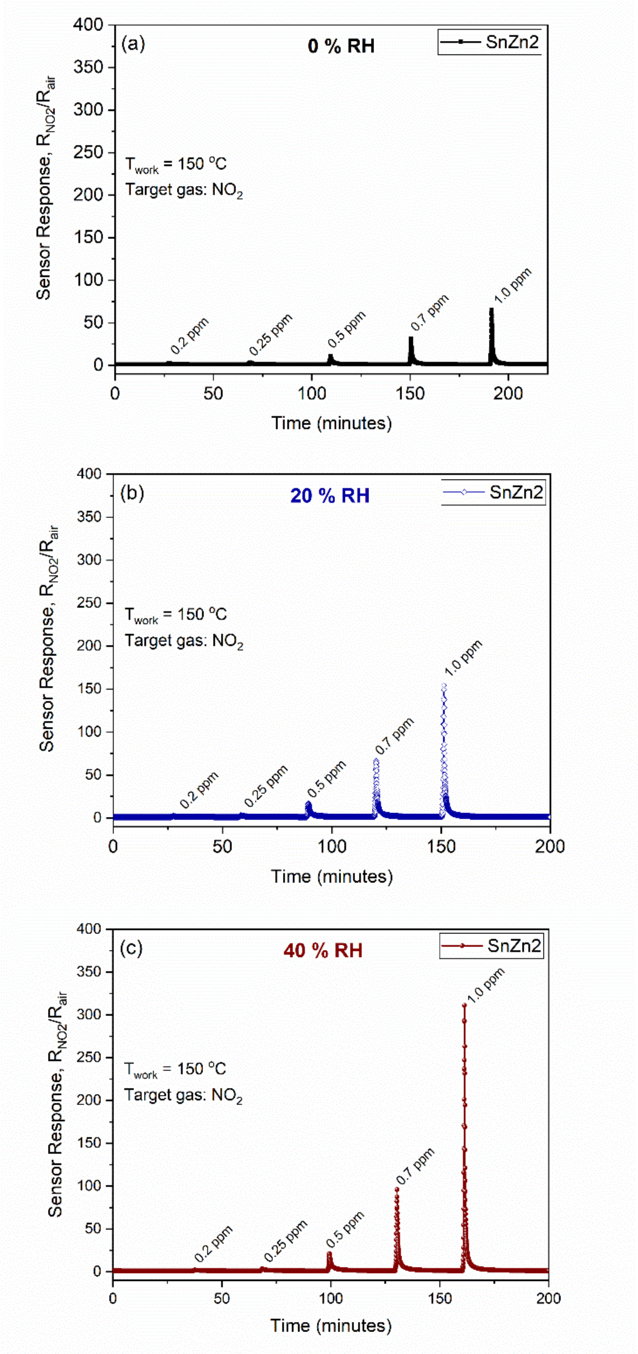

Figure S3. NO_2 gas-sensing performance of SnZn2 nanocrystals kept at 150 °C and exposed to different relative humidity levels. (a) 0 %RH, (b) 20 %RH, and (c) 40 %RH.

Reference

[1] Hilaire, S.; Suess, M.J.; Kranzlin, N.; Bienkowski, K.; Solarska, R.; Augustynski, J.; Niederberger, M. Microwave-assisted nonaqueous synthesis of WO_3 nanoparticles for crystallographically oriented photoanodes for water splitting. *J. Mater. Chem. A* **2014**, *2*, 20530–20537.
